# Association Between Adolescent Violence Exposure and the Risk of Suicide: A 15-Year Study in Taiwan

**DOI:** 10.3390/children12010010

**Published:** 2024-12-24

**Authors:** Chieh Sung, Chi-Hsiang Chung, Chien-An Sun, Chang-Huei Tsao, Daphne Yih Ng, Tsu-Hsuan Weng, Li-Yun Fann, Fu-Huang Lin, Wu-Chien Chien

**Affiliations:** 1 Department of Medical Research, Tri-Service General Hospital, National Defense Medical Center, Taipei 11490, Taiwan; a652667@yahoo.com.tw (C.S.); changhuei@gmail.com (C.-H.T.); a0984295524@gmail.com (T.-H.W.); 2 Department of Senior Citizen Care and Welfare, Deh Yu College of Nursing and Health, Keelung 203, Taiwan; 3 School of Public Health, National Defense Medical Center, Taipei 11490, Taiwan; g694810042@gmail.com (C.-H.C.); noldling@ms10.hinet.net (F.-H.L.); 4 Taiwanese Injury Prevention and Safety Promotion Association, Taipei 11490, Taiwan; 5 Department of Public Health, College of Medicine, Fu-Jen Catholic University, New Taipei City 24205, Taiwan; 040866@mail.fju.edu.tw; 6 Big Data Research Center, College of Medicine, Fu-Jen Catholic University, New Taipei City 24205, Taiwan; 7 Department of Microbiology & Immunology, National Defense Medical Center, Taipei 11490, Taiwan; 8 Department of Family Medicine, Tri-Service General Hospital, National Defense Medical Center, Taipei 11490, Taiwan; conspicuous.com@gmail.com; 9 Graduate Institute of Medical Sciences, National Defense Medical Center, Taipei 11490, Taiwan; 10 Department of Nursing, Taipei City Hospital, Taipei 10684, Taiwan; fanly99@gmail.com

**Keywords:** violence, adolescent suicide, suicide risk, National Health Insurance Research Database (NHIRD)

## Abstract

Background/Objectives: According to the 2023 Ministry of Health and Welfare statistics, the suicide rate among adolescents aged 15 to 24 has steadily increased since 2018, from 3.7 to 5.5 per 100,000 populations, reaching a recent high. Although previous studies have pointed out that the future risk of suicide of those who had suffered from abuse was higher than that of the general population, researchers seldom focused on adolescent groups. Therefore, the aim of this study was to explore the risk of suicide after youth violence and the impact of subsequent comorbid mental illness and suicide risk. Methods: This retrospective matched cohort study analyzed data from the NHIRD, covering the period from 2000 to 2015. A total of 976 cases aged 10–18 who had experienced violence were included in this study. Controlled grouping was conducted by 1:10 matching based on gender, age, and the time of medical treatment, and a control group who had not experienced violence was selected for comparison. We used the Cox proportional hazards model to analyze the risk of suicide among adolescents after exposure to violence. Results: The suicide rate among adolescents who have experienced violence was significantly higher than that of the control group after 15 years of follow-up (1.0% vs. 0.5%). The prevalence of mental illness or disorders in adolescents exposed to violence was significantly higher than in the control group (45.2% vs. 40.1%). Among adolescents who had experienced violence, the methods of suicide included poisoning (solid and liquid) (53.6% vs. 43.2%), hanging (1.2% vs. 0.6%), firearms (2.4% vs. 0%), and cutting instruments (27.4% vs. 22.8%), all of which were significantly higher than in the control group. After adjusting for gender, age, residential area, and mental health comorbidities, the risk of suicide in those who had experienced violence was 1.475 times that of the control group (95% CI = 1.125–1.933; *p* = 0.005). Conclusions: In this study, female, younger age, and comorbid mental disorders were identified as risk factors for suicide among the adolescent victims of violence. Exposure to youth violence was associated with an increased prevalence of emotional disorders, including depression and social isolation, which subsequently elevated the suicide risk. These findings underscore the urgent need for governmental attention to the mental health of adolescent victims of violence. Implementing targeted psychological support and intervention programs could play a crucial role in mitigating the risk of suicide among this vulnerable population.

## 1. Introduction

Adolescent violence remains a pervasive global concern, defined by the physical maltreatment of children by parents or adult household members through acts such as hitting, pushing, choking, shaking, throwing, biting, and burning. These harmful behaviors can lead to bruises or more severe physical injuries. The World Health Organization [1] reports that approximately one-quarter of all adults worldwide have experienced violence during childhood. In the United States, the Department of Health and Human Services estimated that annually between 700,000 and 1.25 million children are subjected to violence or neglect [2]. Similarly, Taiwan’s Ministry of Health and Welfare reported that from 2004 to 2018, between 4000 and 19,000 children experienced violence or neglect each year [3].

Preventing youth suicide is an urgent public health imperative. Suicide is the second leading cause of death among individuals aged 15 to 24, with reported cases of suicidal thoughts and behaviors increasing over the past decade [4,5]. Evidence from self-reported data and clinical assessments indicates that maltreated youth are significantly more likely to contemplate and attempt suicide [6,7]. A recent meta-analysis revealed that young people who have experienced any form of child violence or neglect are 2.91 times more likely to attempt suicide and 2.36 times more likely to experience suicidal ideation compared to their non-maltreated peers [6]. The high prevalence of child maltreatment in the United States amplifies concerns about its impact on youth suicide rates. In 2019, over 3.4 million children were involved in child maltreatment investigations in the U.S. [8]. Furthermore, estimates suggest that 37.4% of U.S. youth will be involved in such investigations by the age of 18 [9]. Globally, the World Health Organization (2020) [10] estimates that approximately one billion children aged two to seventeen experience violence, including child maltreatment, each year. These alarming statistics underscore the critical need for effective suicide prevention strategies targeting maltreated youth worldwide.

However, the relationship between adolescent violence and suicide risk has not been thoroughly investigated. We hypothesized that adolescents who have experienced violence are at a higher risk of future suicide. Therefore, we utilized the National Health Insurance Research Database (NHIRD) to examine whether adolescents exposed to violence were at an increased risk of suicide between 2000 and 2015 in Taiwan.

## 2. Materials and Methods

### 2.1. Data Sources

In this study, we analyzed data from the Taiwan National Health Insurance Research Database (NHIRD) to explore the link between adolescent exposure to violence and the risk of suicide over a 15-year span. Information on adolescent violence incidents was obtained from outpatient and inpatient records in the Taiwan Longitudinal Health Insurance Database for the study duration (2000–2015). The benefits, drawbacks, and specifics of the NHIRD have been discussed in previous studies [11].

The National Health Insurance Program (NHIP) in Taiwan was launched in 1995. As of June 2009, it had contracts with 97% of the nation’s healthcare providers, covering around 23 million beneficiaries—more than 99% of Taiwan’s population [12]. The NHIRD utilizes the International Classification of Diseases, 9th Revision, Clinical Modification (ICD-9-CM) codes to record diagnoses [13].

All diagnoses of adolescent violence were made by pediatricians or emergency medicine physicians based on clinical findings. Furthermore, licensed medical record technicians reviewed and verified the diagnostic codes before the claim for reimbursement to the hospital was approved. According to Taiwan’s Protection of Children and Youths Welfare and Rights Act (2003) [14], clinicians who detect signs or symptoms of child and adolescent violence are mandated to report their findings to the authorized municipal or county agencies within 24 h. Clinicians must exercise meticulous care when diagnosing adolescent violence with the corresponding ICD-9-CM codes to avoid legal repercussions [15]. The diagnoses of psychiatric disorders were made by board-certified psychiatrists, according to the Diagnostic and Statistical Manual of Mental Disorders, Fourth Edition (both original and revised editions) [16].

Additionally, certified medical record technicians review and confirm diagnostic codes before hospital reimbursement claims are processed [14]. The NHIP administration also performs random audits on outpatient records and periodically reviews inpatient claims to maintain diagnostic accuracy [16]. As a result, the data from the NHIRD are regarded as trustworthy. Thus, we used the NHIRD data to examine the relationship between adolescent violence and the occurrence of suicide.

### 2.2. Study Design and Sample

**Figure 1:**
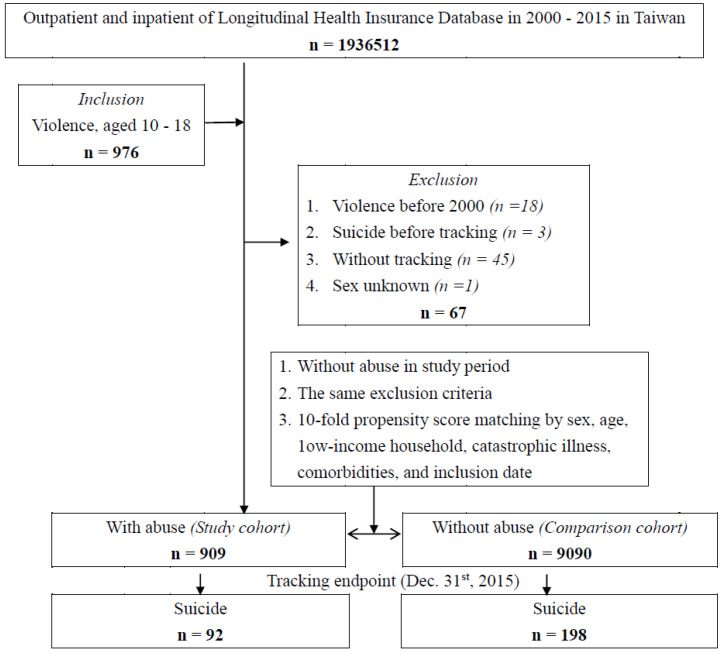
Flowchart of the study.

This study utilized a retrospective matched cohort design. Adolescents diagnosed with violence, between 1 January 2000, and 31 December 2015, were included in the adolescent violence cohort (*n* = 976). In addition, 29,511 controls with no history of adolescent violence during the study period were matched for age, gender, and index year at a ratio of 1:10 to the adolescent violence cohort. Participants over the age of 18 or under 10 years, and those with prior records of adolescent violence or suicide before the index date, were excluded (Figure 1).

### 2.3. Major Outcome Measure

This study aimed to evaluate the association between adolescent violence and the risk of suicide. All participants were followed from 1 January 2000, until the occurrence of suicide, withdrawal from the NHIP, or 31 December 2015.

In this study, the study population includes individuals who have experienced violence (ICD-9-CM codes 955.5 and E967) and suicide events (ICD-9-CM codes E950–E958).

Category 995.5 includes the following: child abuse, unspecified (995.50); child emotional/psychological abuse (995.51); child neglect (995.52); child sexual abuse (995.53); child physical abuse (995.54); shaken infant syndrome (995.55); and other child abuse and neglect (995.59). Category E967 includes perpetrators of child and adult abuse as follows: by father, stepfather, or boyfriend (E967.0); by other specified person (E967.1); by mother, stepmother, or girlfriend (E967.2); by spouse or partner (E967.3); by child (E967.4); by sibling (E967.5); by grandparent (E967.6); by other relative (E967.7); by non-related caregiver (E967.8); and by an unspecified person (E967.9). The date of the first diagnosed adolescent violence case was treated as the index date.

Suicide methods are categorized as follows: solid or liquid substances (E950), domestic gasses (E951), other gasses and vapors (E952), hanging (E953), drowning (E954), firearms (E955), cutting and piercing (E956), jumping (E957), and other unspecified methods (E958). All diagnoses were determined by certified clinicians based on clinical judgment. Additionally, the comorbidities of interest in this population are mental disorders, as identified by ICD-9-CM codes 290–319. All mental disorders diagnoses were made by certified psychiatrists and in accordance with the DSM-V criteria. All detailed ICD codes are provided in Supplemental Table S1.

### 2.4. Variables

Covariates incorporated into the statistical analyses encompassed a comprehensive range of demographic, socioeconomic, and clinical factors. Demographic variables included gender and age, while geographic variables covered the region of residence classified such as northern, central, southern, or eastern Taiwan and the urbanization level of the residential area, stratified into four tiers based on population density and development indicators. The type of healthcare facility accessed by participants was considered, categorized as medical centers, regional hospitals, or local hospitals. Socioeconomic status was evaluated by determining whether participants belonged to low-income households. Clinical covariates included the presence of catastrophic illnesses and mental disorders, identified through the relevant diagnostic codes. Additionally, the season during which data were collected, spring, summer, autumn, or winter, was included to control for potential seasonal variations affecting health outcomes. The urbanization level was defined according to the population size and the various indicators of development. Urbanization level 1 was defined as areas with a population of over 1,250,000 inhabitants with a specific designation of political, economic, cultural, and metropolitan development. Urbanization level 2 was defined as areas with a population between 500,000 and 1,249,999 inhabitants, playing an important role in the political system, economy, and culture. Urbanization levels 3 and 4 were defined as areas with populations of 149,999–499,999 and less than 149,999 inhabitants, respectively [17]. Comorbidities included mental disorders. The Charlson comorbidity index (CCI) is one of the most widely used comorbidity indexes [18,19], which consists of 22 conditions [20]. The score was calculated based on the presence of the relevant comorbidities (according to the ICD-9-CM codes) [21], with a score of zero indicating the absence of comorbidities and higher scores indicating a higher comorbidity burden [22].

### 2.5. Statistical Analysis

All statistical analyses were conducted using SPSS software version 29 (SPSS Inc., Chicago, IL, USA). Chi-square (χ^2^) and Mann–Whitney U test were employed to assess the distributions of categorical and continuous variables, respectively. Fisher’s exact test was used to assess differences between the two cohorts with respect to categorical variables. The associations between time-to-event outcomes and clinical characteristics were examined using the Kaplan–Meier method and multivariate Cox regression analysis with stepwise selection; the results are reported as hazard ratios (HR) and 95% confidence intervals (CI). Adjustments were made for age, gender, and covariates for inclusion in the multivariate model. Bonferroni correction for multiple comparisons was performed. A two-tailed Bonferroni-corrected *p* value < 0.05 was regarded as statistically significant.

The factors influencing the different suicide subgroups were analyzed using Cox proportional hazards regression models. A Bonferroni correction was applied to adjust for multiple comparisons. The Cox model estimates hazard ratios for each factor while controlling for potential confounders. The Bonferroni correction adjusts *p*-values by dividing the significance level by the number of comparisons to control for type I error. The results are presented as follows for each subgroup: solid or liquid substances, gasses in domestic use, other gasses and vapors, hanging, drowning, firearms, cutting and piercing, jumping, and others.

### 2.6. Ethics Approvals

This research was carried out in compliance with the World Medical Association’s Code of Ethics (Declaration of Helsinki). This study was approved by the Institutional Review Board of Tri-Service General Hospital at the National Defense Medical Center in Taipei, Taiwan, and the requirement of individual consent was waived because all identifying data were encrypted (TSGHIRB No. B202405024).The NHIRD is a publicly available database that contains depersonalized patient information to ensure patient anonymity.

## 3. Results

### 3.1. Baseline Characteristics

The demographic and clinical characteristics of both groups are summarized in Table 1. We identified 909 adolescents with a documented history of violence and selected 9090 matched controls without such a history. The mean age of adolescents in the violence cohort was 14.18 ± 4.72 years, with a higher proportion of females than males. Overall, the sample was predominantly female (97.47%), with only 2.53% of participants identifying as male. Furthermore, the distribution of gender remained virtually identical across both the violence and non-violence cohorts, each consisting of 2.53% males and 97.47% females. Notably, significant differences between the violence and control cohorts were observed in terms of geographical location and urbanization levels.

### 3.2. Characteristics of the Study Population at Endpoint

By the end of the study period, 92 out of the 909 adolescents who had experienced violence (10.12%) died by suicide, compared to 198 out of the 9090 individuals in the control group (2.18%), demonstrating a statistically significant difference (*p* < 0.001; Table 2). Significant disparities were observed between the violence-exposed and control groups when comparing geographical location and urbanization level. In contrast, there were no significant differences between the two groups regarding gender, age, low-income household status, presence of catastrophic illnesses, mental disorders, CCI scores, season, or the level of care. The comprehensive data are presented in Table 2.

### 3.3. Risk of Suicide According to Adolescent Violence Exposure

**Figure 2:**
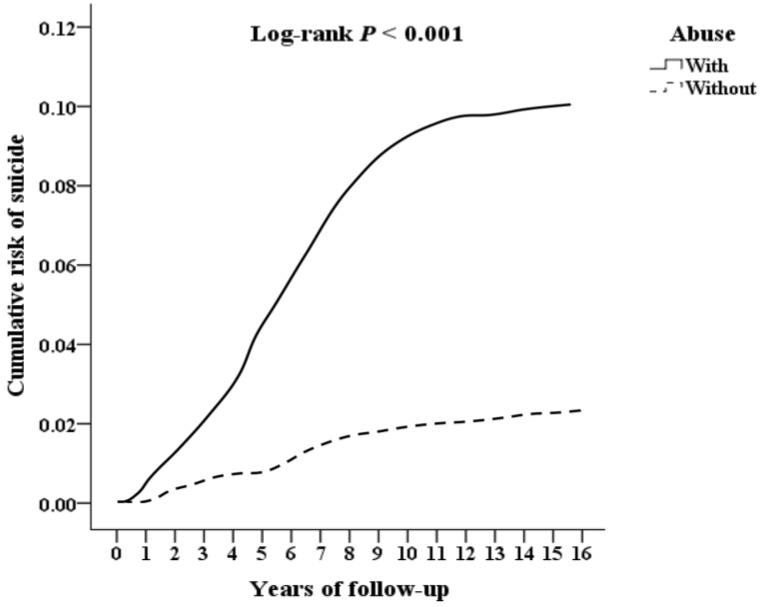
Kaplan–Meier for cumulative risk of suicide aged 10–18, stratified by violence with log-rank test.

The Kaplan–Meier survival analysis revealed that adolescents with a history of violence had a significantly higher cumulative incidence of suicide over the 15-year follow-up period compared to the matched control group (log-rank test, *p* < 0.001; Figure 2). The results from Tables S2 and S3 show no significant difference in follow-up years between adolescents with and without a history of violence (*p* > 0.05). However, in terms of years to suicide, there was a significant difference between the two groups, with a *p*-value of 0.031, suggesting that exposure to violence may lead to an earlier occurrence of suicide. These findings indicate that while exposure to violence does not affect the length of follow-up, it significantly impacts the age at which suicide occurs. This underscores the importance of early intervention for adolescents who have experienced violence to reduce suicide risk. The relationship between gender, violence, and psychological behavior is complex and warrants further research.

### 3.4. Factors of Suicide Using Cox Regression

Table 3 presents the results of the Cox proportional hazards regression analysis examining factors associated with suicide risk among adolescents who have experienced violence. The unadjusted hazard ratio (HR) for suicide in the violence cohort was 1.787 (95% CI: 1.246–2.033; *p* < 0.001), indicating a significantly elevated risk compared to the control group. After adjusting for multiple covariates, including gender, age group, low-income household status, presence of catastrophic illness, mental disorders, CCI score, season, geographic location, urbanization level, and the level of care, the association remained significant. The adjusted hazard ratio (aHR) was 1.592 (95% CI: 1.137–1.993; *p* < 0.001), suggesting that adolescent violence is independently associated with an increased risk of suicide. Several covariates were also significantly correlated with suicide risk. The crude HR for females was 2.098 (95% CI: 1.358–2.886, *p* < 0.001), indicating that prior to adjusting for other factors, the risk of suicide among females was approximately twice that of males. After adjusting for potential confounders, the aHR remained statistically significant at 1.523 (95% CI: 1.072–1.831, *p* = 0.012), suggesting that females continue to exhibit a significantly higher risk of suicide compared to males, even after accounting for other influencing variables. Additionally, the presence of mental disorders and higher levels of care were associated with an increased incidence of suicide (*p* < 0.05).

### 3.5. Factors of Suicide Stratified by Variables Listed in the Table Using Cox Regression and Bonferroni Correction for Multiple Comparisons

The patients were stratified by the variables presented in Table 3, and the adjusted hazard ratios of the different subgroups were calculated (Table 4). Over the course of the study, adolescents who had experienced violence exhibited 92 suicide events over 7074.36 person-years (PYs) of observation, resulting in an incidence rate of 1300.47 per 100,000 PYs. In contrast, the control group encountered 198 suicide events over 69,894.12 PYs, corresponding to an incidence rate of 283.29 per 100,000 PYs. After applying the Bonferroni correction for multiple comparisons, the risk of suicide was significantly higher among adolescents with a history of violence compared to those without such a history. The aHR was 1.592 (95% CI: 1.137–1.993; *p* < 0.001), indicating that adolescents affected by violence had nearly a 1.5 times increased risk of suicide. When stratified by gender, male exposure to violence showed a significantly elevated suicide risk (aHR = 1.500, 95% CI: 1.071–1.872, *p* = 0.015). Among females, the risk was even higher (aHR = 1.596, 95% CI: 1.401–1.998, *p* < 0.001). These findings underscore the heightened impact of violence on suicide risk, particularly among females, highlighting the need for targeted interventions for this high-risk group. Notably, the presence of mental disorders and higher levels of care were significantly associated with an increased incidence of suicide, the aHR was 2.369 (95% CI: 2.369–2.972; *p* < 0.001).

### 3.6. Factors of Suicide Subgroups Using Cox Regression and Bonferroni Correction for Multiple Comparisons

Adolescents exposed to violence exhibited a significantly elevated risk of suicide across various methods. Specifically, the adjusted hazard ratios (AHRs) for different suicide methods were as follows: ingestion of solids or liquids (AHR = 1.607), exposure to other gasses and vapors (AHR = 1.714), hanging (AHR = 2.058), cutting and piercing (AHR = 1.656), and jumping (AHR = 1.523) (Table 5).

### 3.7. Factors of Suicide Stratified by Violence and Mental Disorders Using Cox Regression

**Figure 3:**
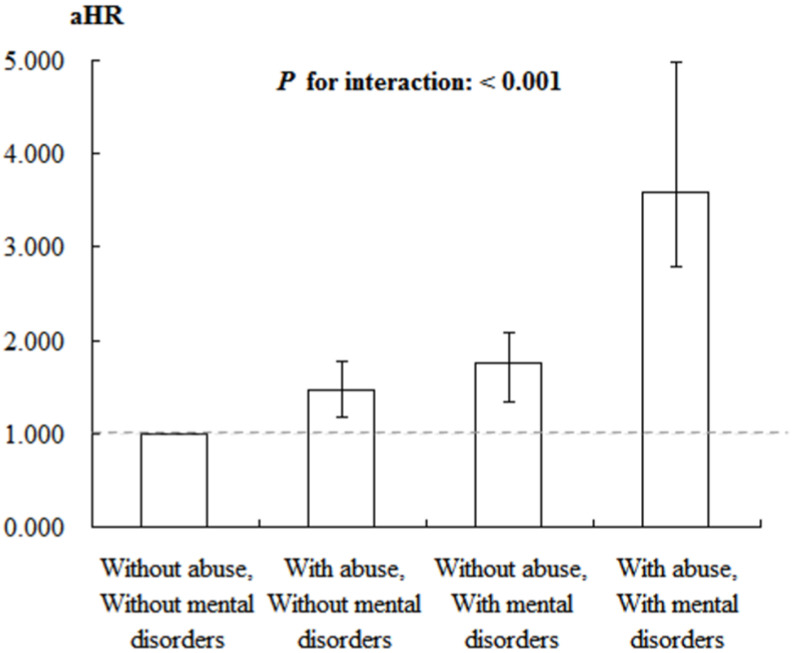
Joint effect for factors of suicide stratified by violence and mental disorders using Cox regression.

The analysis of suicide risk factors using the Cox regression model indicates a significant impact of both violence and mental disorders on suicide risk. In the reference group, which included individuals without a history of violence or mental disorders, the aHR was set at 1.000. In comparison, individuals without a history of violence but with mental disorders exhibited a significantly elevated risk of suicide, with an aHR of 1.465 (95% CI: 1.172–1.779, *p* < 0.001). Among those who experienced violence but did not have mental disorders, the suicide risk further increased, with an aHR of 1.756 (95% CI: 1.340–2.075, *p* < 0.001). Furthermore, individuals who experienced both violence and had mental disorders showed the highest risk of suicide, with an aHR of 3.586 (95% CI: 2.781–4.986, *p* < 0.001). Additionally, the interaction between violence and mental disorders was significant, as indicated by the *p* for interaction value (*p* < 0.001), highlighting a notable synergistic effect that further elevated the risk of suicide (Table 6, Figure 3).

## 4. Discussion

This study examined the relationship between adolescent violence and suicidal behaviors, assessing whether all forms of adolescent violence were associated with an increased risk of suicidality in both univariate logistic regression models and multivariable logistic regression models that controlled for covariates. In our study, we found that the suicide rate of adolescents who have experienced violence was significantly higher than that of the control group (10.12% vs. 2.18%; *p* < 0.001), with an aHR of 1.592 after 15 years of observation. The result is consistent with Zygo et al. [23], which demonstrated that psychological, physical violence, and family violence were all risk factors not only for suicide ideation but also for suicide attempt and even suicide death. The findings of this study align with those of numerous other investigations [24,25,26]. However, an alternative study suggests that emotional violence is the most significant predictor of suicide attempts, with physical violence following closely behind [27]. Several theories may account for the relationship between adolescent violence and suicidality. The Schematic Appraisals Model for Suicide posits that negative childhood experiences can foster a growing sense of self-defeat, ultimately leading individuals to perceive suicidality as an escape mechanism [28,29]. In a study from America [23], the peak age of suicide in rural or urban area was 16 to 18 years old, which was consistent with our research. Both the studies reminded us to closely monitor and observe the mental and psychologic status of the youths in this period.

Thus, concerns and caution should rise keenly for the adolescents in some situations, such as unusual wounds or bruises, rapid mood change, weird behavior, abnormal vaginal bleeding, and relationship with schoolmates. The methods and tools for adolescent suicide may be different in distinct countries or cultures. In our study, the most common methods were the consumption of liquid or solid components in both the group with/without being the victims of violence, the method of suicide is self-hanging, followed by firearms, self-burning, and self-poisoning. Thus, it is important for different countries to formulate policies exclusively, such as gun control, chemicals restriction for less accessibility, or the prohibition of entrance to the top floor in tall buildings [24,25]. In addition, with the development of technology, electronic bullying is worth being noticed. According to the study by [30] with data collected by the Centers for Disease Control and Prevention in America, not only physical bullying but also electronic bullying increased the risk for suicide ideation and attempt, pointing out the importance of education of internet politeness and online social contact for youth. The risk factors for suicide of adolescence and youth included history of violence, female, young age (from 10 to 24 years old), psychological disorders, depression, and substance use in Taiwan, which were similar to the results in other regions around the world [25,30,31,32,33,34,35,36], indicating a somewhat biophysical mechanism that crosses ethnics. However, in a study in America [30], Asian adolescents are more prone to having suicide ideation and attempts than other ethnics, making it important for Asian educators and family members to pay more attention to their adolescents, including Taiwan.

In terms of gender differences, the statistical results of this study suggest that female adolescents experience higher rates of violence and suicide-related behaviors compared to their male counterparts. Previous research has shown that girls are more likely to encounter general violence than boys, particularly sexual harassment, which is often more difficult to identify in clinical settings compared to physical violence [31]. Additionally, studies by Canetto and Sakinofsky [32] and Conner and Duberstein [33] have indicated that female adolescents are at greater risk of experiencing suicidal ideation and attempts when compared to male peers. This could help explain the higher suicide rates observed among females relative to males. The gender disparity observed in this study may also be influenced by the broader age range of the adolescent population under investigation. However, our findings contrast with those from a hospital-based study conducted in the United States, which found that physical violence in children was more frequently identified in boys than in girls within the medical system [34]. Furthermore, research by Farrell, Petros, and Hawkins [35], using data from the Centers for Disease Control and Prevention’s Compressed Mortality Files, revealed a male-dominant cohort in childhood violence. Similarly, Ashraf, Kahn, and Hussain [34] reported that male adolescents reported significantly higher levels of violence than their female counterparts in community settings. In contrast, studies by Kolev, Petrova, and Vassilev [37] and Roh, Park, and Kim [38] found that suicide rates among males were higher than those observed in females. These findings highlight the multifaceted and complex role gender plays in suicidal behavior and experiences of violence. These differences in research outcomes may be potentially influenced by societal perceptions of physical discipline or the way physicians interpret male children’s behavior. This, in turn, may impact diagnostic accuracy, suggesting the need for further research to explore the underlying causes and the potential for targeted interventions.

This study has several limitations. First, the potential for selection bias cannot be ruled out. The incidence of adolescent violence may be significantly underestimated; as medical attention is typically sought only for adolescents who experience more severe forms of maltreatment. This issue is further compounded by cases where the abuser is a parent or caregiver, reducing the likelihood of the adolescent seeking medical evaluation. Additionally, the diagnoses of adolescent violence were based solely on clinical judgment and experience, which lack standardized criteria and may result in inaccuracies or underdiagnoses. The reliance on medical evaluations also poses a limitation, as prior studies (e.g., adverse childhood experience data) suggest that the prevalence of interfamilial physical violence is much higher than what is reflected in medical reports. Consequently, some individuals in the control group may have also been exposed to violence, potentially biasing the analysis.

Second, there may be gender-based biases in identifying violence-related injuries. Violence against male adolescents is more likely to be overlooked or misinterpreted as normal behavior. In some cases, physical discipline towards male adolescents may be perceived as acceptable rather than abusive, leading to the underreporting of such incidents in the NHIRD.

Third, this study was limited by the data available in the health insurance database. We were unable to consider other important factors that may influence the risk of adolescent violence, such as the parent–child relationship, marital status, educational background, or religious beliefs. Additionally, the NHIRD did not provide clinical biochemistry data, the Glasgow Coma Scale [39], or abbreviated injury severity scores [40], which could have provided additional insights.

Fourth, privacy concerns and data protection protocols prevented direct interaction with patients, limiting access to additional information such as their mental status. Furthermore, as this study relied exclusively on inpatient data, cases involving minor injuries, outpatient care, or emergency visits were not captured. As a result, the study’s findings may be biased toward more severe injury cases.

Lastly, in compliance with the regulations outlined by the Health and Welfare Data Center (HWDC) under the Ministry of Health and Welfare (MOHW) of Taiwan, it is not feasible to acquire new analytical results in the short term. Even with the potential availability of additional data, it is unlikely to significantly impact the trajectory of our findings. The primary objective of this study is to examine trends in suicide among victims of adolescent violence in Taiwan over an extended period. Notably, the dataset employed encompasses a sample of 2 million individuals, representing a subset of Taiwan’s 23 million population, which is sufficiently large to offer a reliable reflection of broader societal trends.

Violence is caused by the presence of multiple risk factors and a combination of very few protective factors. Violence can be prevented by reducing risk factors and strengthening protective factors. Conducting this requires comprehensive policies that form part of a so-called “integrated approach” to violence prevention, in other words, an overall strategy that depends on the cooperation of many different sectors.

## 5. Conclusions

In this study, adolescents who experienced violence had a significantly higher risk of future suicide compared to the control group, with the suicide rate increasing by up to 1.592 times. Notably, among those with comorbid mental disorders, the suicide risk rose to 2.369 times that of the control group. Exposure to youth violence may lead to emotional disorders, including depression and social isolation, which subsequently elevate the risk of suicide. Exposure to youth violence can lead to emotional disorders, including depression and social isolation, which in turn elevate the risk of suicide. To reduce the occurrence of suicide, it is crucial to not only provide psychological support and pay closer attention to the mental health of youth violence victims but also for the government to implement more practical measures. There is an urgent need to emphasize that experiencing violence or abuse is not shameful. Victims should seek medical attention or obtain protection and support from the government or healthcare institutions as early as possible to reduce their sense of guilt and prevent the loss of critical opportunities for timely intervention, documentation, and treatment. Additionally, schools and workplaces must establish comprehensive programs for gender equality education, bullying prevention, and related awareness initiatives. Third-party reporting and support mechanisms should also be put in place to ensure that victims do not fear being unable to continue functioning within their communities, which may otherwise lead them to endure in silence and ultimately result in tragic outcomes.

## Figures and Tables

**Table 1:** Characteristics of study in the baseline.

Violence	Total		With		Without		*p*
Variables	*n*	%	*n*	%	*n*	%	
Total	9999		909		9090		
gender							0.999
Male	253	2.53	23	2.53	230	2.53	
Female	9746	97.47	886	97.47	8860	97.47	
Age (years)	14.28 ± 4.85		14.18 ± 4.72		14.29 ± 4.86		0.314
Low-income household							0.948
Without	7405	74.06	674	74.15	6731	74.05	
With	2594	25.94	235	25.85	2359	25.95	
Catastrophic illness							0.704
Without	8871	88.72	803	88.34	8068	88.76	
With	1128	11.28	106	11.66	1022	11.24	
Mental disorders							0.999
Without	9460	94.61	860	94.61	8600	94.61	
With	539	5.39	49	5.39	490	5.39	
CCI	0.74 ± 0.68		0.72 ± 0.65		0.74 ± 0.68		0.107
season							0.999
Spring (Mar–May)	2398	23.98	218	23.98	2180	23.98	
Summer (Jun–Aug)	2673	26.73	243	26.73	2430	26.73	
Autumn (Sep–Nov)	2552	25.52	232	25.52	2320	25.52	
Winter (Dec–Feb)	2376	23.76	216	23.76	2160	23.76	
Location							<0.001
Northern Taiwan	3500	35.00	375	41.25	3125	34.38	
Middle Taiwan	2546	25.46	234	25.74	2312	25.43	
Southern Taiwan	2358	23.58	251	27.61	2107	23.18	
Eastern Taiwan	1072	10.72	49	5.39	1023	11.25	
Outlets islands	523	5.23	0	0.00	523	5.75	
Urbanization level							<0.001
1 (The highest)	2922	29.22	298	32.78	2624	28.87	
2	3421	34.21	346	38.06	3075	33.83	
3	1638	16.38	112	12.32	1526	16.79	
4 (The lowest)	2018	20.18	153	16.83	1865	20.52	
Level of care							0.701
Hospital center	5252	52.53	489	53.80	4763	52.40	
Regional hospital	3331	33.31	297	32.67	3034	33.38	
Local hospital	1416	14.16	123	13.53	1293	14.22	

*p*: Chi-square/Fisher’s exact test on category variables and U-test on continuous variables.

**Table 2:** Characteristics of study at the endpoint.

Violence	Total		With		Without		*p*
Variables	*n*	%	*n*	%	*n*	%	
Total	9999		909		9090		
Suicide							<0.001
Without	9709	97.10	817	89.88	8892	97.82	
With	290	2.90	92	10.12	198	2.18	
Gender							0.999
Male	253	2.53	23	2.53	230	2.53	
Female	9746	97.47	886	97.47	8860	97.47	
Age (yrs)	21.99 ± 9.96		21.95 ± 9.86		22.00 ± 9.97		0.763
Low-income household							0.948
Without	7401	74.02	672	73.93	6729	74.03	
With	2598	25.98	237	26.07	2361	25.97	
Catastrophic illness							0.683
Without	8863	88.64	802	88.23	8061	88.68	
With	1136	11.36	107	11.77	1029	11.32	
Mental disorders							0.707
Without	9454	94.55	857	94.28	8597	94.58	
With	545	5.45	52	5.72	493	5.42	
CCI	0.75 ± 0.69		0.74 ± 0.67		0.75 ± 0.69		0.310
Season							0.873
Spring	2415	24.15	226	24.86	2189	24.08	
Summer	2673	26.73	247	27.17	2426	26.69	
Autumn	2547	25.47	230	25.30	2317	25.49	
Winter	2364	23.64	206	22.66	2158	23.74	
Location							<0.001
Northern Taiwan	3411	34.11	372	40.92	3039	33.43	
Middle Taiwan	2550	25.50	246	27.06	2304	25.35	
Southern Taiwan	2341	23.41	223	24.53	2118	23.30	
Eastern Taiwan	1163	11.63	59	6.49	1104	12.15	
Outlets islands	534	5.34	9	0.99	525	5.78	
Urbanization level							<0.001
1 (The highest)	2934	29.34	299	32.89	2635	28.99	
2	3374	33.74	342	37.62	3032	33.36	
3	1695	16.95	117	12.87	1578	17.36	
4 (The lowest)	1996	19.96	151	16.61	1845	20.30	
Level of care							0.700
Hospital center	5235	52.36	483	53.14	4752	52.28	
Regional hospital	3324	33.24	291	32.01	3033	33.37	
Local hospital	1440	14.40	135	14.85	1305	14.36	

*p*: Chi-square/Fisher exact test’s on category variables and U-test on continuous variables.

**Table 3:** Factors of suicide using Cox regression.

Variables	Crude HR	95% CI	95% CI	*p*	aHR	95% CI	95% CI	*p*
Violence								
Without	Reference				Reference			
With	1.787	1.246	2.033	<0.001	1.592	1.137	1.993	<0.001
Gender								
Male	Reference				Reference			
Female	2.098	1.358	2.886	<0.001	1.523	1.072	1.831	0.012
Age (yrs)	0.894	0.589	1.182	0.486	0.971	0.632	1.289	0.570
Low-income household								
Without	Reference				Reference			
With	2.303	1.493	3.701	<0.001	1.572	1.099	1.977	0.001
Catastrophic illness								
Without	Reference				Reference			
With	1.862	1.198	2.596	<0.001	1.303	1.050	1.684	0.024
Mental disorders								
Without	Reference				Reference			
With	2.979	1.865	4.228	<0.001	2.666	1.356	3.784	<0.001
CCI	1.201	1.186	1.277	<0.001	1.142	1.063	1.225	0.018
Season								
Spring	Reference				Reference			
Summer	1.779	1.287	2.074	<0.001	1.440	1.088	1.720	0.006
Autumn	1.933	1.483	2.335	<0.001	1.518	1.165	1.836	<0.001
Winter	1.502	1.095	1.982	0.003	1.386	1.024	1.689	0.037
Location								
Northern Taiwan	Reference				Multicollinearity with urbanization level			
Middle Taiwan	0.842	0.301	1.378	0.672	Multicollinearity with urbanization level			
Southern Taiwan	0.986	0.389	1.460	0.573	Multicollinearity with urbanization level			
Eastern Taiwan	0.597	0.284	0.997	0.048	Multicollinearity with urbanization level			
Outlets islands	0.732	0.042	196.678	0.925	Multicollinearity with urbanization level			
Urbanization level								
1 (The highest)	2.135	1.487	2.865	<0.001	1.795	1.194	2.512	<0.001
2	1.911	1.374	2.784	<0.001	1.783	1.113	2.505	<0.001
3	1.506	1.010	1.701	0.045	1.322	0.807	1.571	0.189
4 (The lowest)	Reference				Reference			
Level of care								
Hospital center	2.785	2.013	3.389	<0.001	2.106	1.370	2.864	<0.001
Regional hospital	1.843	1.562	2.131	<0.001	1.442	1.115	1.765	<0.001
Local hospital	Reference				Reference			

HR = hazard ratio, CI = confidence interval, and aHR = adjusted HR: Adjusted variables are listed in the table.

**Table 4:** Factors of suicide stratified by variables listed in the table using Cox regression and Bonferroni correction for multiple comparisons.

Violence	With			Without (Reference)			With vs. Without (Reference)			
Stratified	Events	PYs	Rate	Events	PYs	Rate	aHR	95% CI	95% CI	*p*
Total	92	7074.36	1300.47	198	69,894.12	283.29	1.592	1.137	1.993	<0.001
gender										
Male	3	175.23	1712.04	7	1768.33	395.85	1.500	1.071	1.872	0.015
Female	89	6899.13	1290.02	191	68,125.79	280.36	1.596	1.401	1.998	<0.001
Low-income household										
Without	67	5230.01	1281.07	146	51,734.81	282.21	1.574	1.121	1.963	<0.001
With	25	1844.35	1355.49	52	18,159.31	286.35	1.642	1.178	2.059	<0.001
Catastrophic illness										
Without	79	6236.10	1266.82	175	61,981.84	282.34	1.556	1.113	1.949	<0.001
With	13	838.26	1550.83	23	7912.28	290.69	1.852	1.327	2.320	<0.001
Mental disorders										
Without	84	6669.75	1259.42	187	66,103.87	282.89	1.542	1.104	1.913	<0.001
With	8	404.61	1977.21	11	3790.25	290.22	2.369	1.688	2.972	<0.001
Season										
Spring	20	1758.82	1137.13	45	16,831.25	267.36	1.475	1.053	1.849	0.022
Summer	27	1992.33	1355.20	53	18,653.31	284.13	1.656	1.180	2.072	<0.001
Autumn	26	1789.14	1453.21	54	17,815.09	303.11	1.667	1.188	2.085	<0.001
Winter	19	1534.07	1238.54	46	16,594.47	277.20	1.543	1.104	1.940	<0.001
Urbanization level										
1 (The highest)	32	2323.02	1377.52	59	20,262.43	291.18	1.641	1.177	2.059	<0.001
2	35	2661.67	1314.96	66	23,313.17	283.10	1.611	1.152	2.023	<0.001
3	11	908.41	1210.91	34	12,133.66	280.21	1.498	1.070	1.878	0.015
4 (The lowest)	14	1181.26	1185.18	39	14,184.86	274.94	1.494	1.067	1.870	0.017
Level of care										
Hospital center	52	3758.98	1383.35	107	36,538.04	292.85	1.647	1.173	2.066	<0.001
Regional hospital	28	2253.18	1242.69	64	23,231.55	275.49	1.562	1.118	1.982	<0.001
Local hospital	12	1062.20	1129.73	27	10,124.53	266.68	1.460	1.049	1.842	0.026

PYs = person-years, Rate: per 100,000 PYs, and aHR = adjusted hazard ratio, adjusted for the variables listed in Table 3. CI = confidence interval.

**Table 5:** Factors of suicide subgroups using Cox regression and Bonferroni correction for multiple comparisons.

Violence	With	Without (Reference)	With vs. Without (Reference)			
Suicide	Events	Events	aHR	95% CI	95% CI	*p*
Overall	92	198	1.592	1.137	1.993	<0.001
Solid or liquid	38	81	1.607	1.148	2.012	<0.001
Gasses in domestic use	0	2	0.000	-	-	0.999
Other gasses and vapors	12	24	1.714	1.223	2.145	<0.001
Hanging	3	5	2.058	1.468	2.571	<0.001
Drowning	0	2	0.000	-	-	0.999
Firearms	1	0	∞	-	-	0.997
Cutting and piercing	29	60	1.656	1.172	2.073	<0.001
Jumping	4	9	1.523	1.081	1.909	0.009
Others	5	15	1.142	0.823	1.424	0.157

aHR = adjusted hazard ratio, adjusted for the variables listed in Table 3. CI = confidence interval.

**Table 6:** Factors of suicide stratified by violence and mental disorders using Cox regression.

Violence	Mental Disorders	aHR	95% CI	95% CI	*p*	*p* for Interaction
Without	Without	1.000				<0.001
With	Without	1.465	1.172	1.779	<0.001	
Without	With	1.756	1.340	2.075	<0.001	
With	With	3.586	2.781	4.986	<0.001	

aHR = adjusted hazard ratio, adjusted for the variables listed in Table 3. CI = confidence interval.

## Data Availability

This study uses third-party data. Taiwan launched a single-payer National Health Insurance program on 1 March 1995. The database of this program contains registration files and original claim data for reimbursement. Large, computerized databases were derived from this system by the National Health Insurance Administration. Investigators interested may submit a formal proposal to NHIRD (https://dep.mohw.gov.tw/DOS/cp-5119-59201-113.html, accessed on 10 December 2024) The authors confirm that they did not have any special access privileges.

## Supplementary Materials

The following supporting information can be downloaded at the following website: https://www.mdpi.com/article/10.3390/children12010010/s1, Table S1. Abbreviation, ICD-9-CM, and definition; Table S2. Years of follow-up; Table S3. Years to suicide.
