# Supplementary material for: Association Between Adolescent Violence Exposure and the Risk of Suicide: A 15-Year Study in Taiwan
Source: Children (Basel). 2024 Dec 24;12(1):10. doi: 10.3390/children12010010 (PMC11763419; doi:10.3390/children12010010)
Supplement: Supplementary file 1 [file children-12-00010-s001.zip › children-3367305-supplementary.pdf]

children

MDPI

Supplementary Materials

Table S1. Abbreviation, ICD-9-CM, and definition

Definition	ICD-9-CM
**Study population:** Abuse	**955.5, E967**
**Child maltreatment syndrome**	**995.5**
Child abuse, unspecified	995.5
Child emotional/psychological abuse	995.51
Child neglect (nutritional)	995.52
Child sexual abuse	995.53
Child physical abuse	995.54
Shaken infant syndrome	995.55
Other child abuse and neglect	995.59
**Perpetrator child and adult abuse**	**E967**
Perpetrator child and adult abuse by father, stepfather or boyfriend	E967.0
Perpetrator child and adult abuse by other specified person	E967.1
Perpetrator child and adult abuse by mother, stepmother or girlfriend	E967.2
Perpetrator child and adult abuse by spouse or partner	E967.3
Perpetrator child and adult abuse by child	E967.4
Perpetrator child and adult abuse by sibling	E967.5
Perpetrator child and adult abuse by grandparent	E967.6
Perpetrator child and adult abuse by other relative	E967.7
Perpetrator child and adult abuse by non- related caregiver	E967.8
Perpetrator child and adult abuse by unspecified person	E967.9
**Events: Suicide**	E950 - E958
Solid or liquid	E950
Gases in domestic use	E951
Other gases and vapors	E952
Hanging	E953
Drowning	E954
Firearms	E955
Cutting and piercing	E956
Jumping	E957
Others	E958
**Comorbidities:**	
Mental disorders	290 - 319
Charlson comorbidity index(CCI)	

Table S2. Years of follow-up

Violence	Min	Median	Max	Mean ± SD	*P*
With	0.02	7.34	15.72	7.78 ± 6.52	
Without	0.02	7.30	15.70	7.69 ± 6.68	
Total	0.02	7.32	15.72	7.70 ± 6.67	0.422

***P*: U-test**

Table S3. Years to suicide

Violence	Min	Median	Max	Mean ± SD	*P*
With	0.02	5.02	15.68	6.01 ± 6.38	
Without	0.03	5.29	15.69	6.48 ± 6.72	
Total	0.02	5.15	15.69	6.44 ± 6.69	0.031

***P*: U-test**
